# An Improved Synchrosqueezing S-Transform and Its Application in a GPR Detection Task

**DOI:** 10.3390/s24102981

**Published:** 2024-05-08

**Authors:** Hongqiang Xiong, Baizhou An, Boyang Sun, Jiayu Lu

**Affiliations:** 1 College of Geo-Exploration Science and Technology, Jilin University, Changchun 130026, China; 2 Ningxia Geophysical and Geochemical Exploration Institute (Autonomous Regional Deep Earth Exploration Center), Yinchuan 750001, China; 3 School of Resource and Geosciences, China University of Mining and Technology, Xuzhou 221000, China

**Keywords:** ground penetrating radar, tunnel inspection, S-transform, synchrosqueezing

## Abstract

The S-transform is a fundamental time–frequency (T-F) domain analysis method in ground penetrating radar (GPR) data processing and can be used for identifying targets, denoising, extracting thin layers, and high-resolution imaging. However, the S-transform spectrum experiences energy leakage near the instantaneous frequency. This phenomenon causes frequency components to erroneously spread over a wider range, impacting the accuracy and precision of GPR data processing. Synchrosqueezing is an effective method to prevent spectrum leakage. In this work, we introduce the synchrosqueezing generalized phase-shifting S-transform (SS-GPST). Initially, it resolves the compatibility issue between the S-transform and the synchrosqueezing strategy through phase-shifting. Subsequently, the SS-GPST accomplishes spectral energy focusing and resolution enhancement via a generalized parameter and synchrosqueezing. A synthetic signal test shows that the SS-GPST excels over other methods at focusing degree, spectral resolution, and signal reconstruction accuracy and speed. In actual GPR tunnel detection data processing, we assess the adaptability of the SS-GPST from three aspects: spectral energy distribution, thin layer identification, and data denoising. The results indicate: (1) compared to other methods, the SS-GPST accurately expresses spectral components with a strong focusing degree and fewer interference components; (2) high-frequency slices of the SS-GPST accurately detect the top and bottom interfaces of a 3.0–3.5 cm reinforcement protection layer; and (3) due to fewer interference components in the SS-GPST spectrum, reconstructing GPR profiles through the SS-GPST inverse transform is an efficient denoising technique. The SS-GPST demonstrates adaptability to different data processing purposes, offers high-resolution T-F spectra, and shows potential to supersede the S-transform.

## 1. Introduction

Time–frequency (T-F) transforms decompose signals into the T-F domain, illuminating the distribution patterns and structural characteristics of various components. The most commonly used T-F transforms include the short-time Fourier transform (STFT) [1], the continuous wavelet transform (CWT) [2], and the S-transform (ST) [3]. In ground penetrating radar (GPR), high-frequency electromagnetic waves attenuate rapidly in media, resulting in weak effective signal energy. Consequently, the ST, which is sensitive to high-frequency, low-amplitude signals, finds widespread application in GPR data processing.

For example, Li et al. [4] employed the ST to detect human targets and ascertain life-sign frequencies. Szymczyk et al. [5] proposed a three-dimensional ST for identifying sinkholes in geological structures. Riba et al. [6] applied the ST to enhance the signal-to-noise ratio in 3D GPR data for archaeological exploration. Zhang et al. [7] utilized generalized ST slices to identify thin layers in lakebed sediment. Further, Li et al. [8] achieved high-resolution imaging of the lunar shallow subsurface through the ST. However, the spectral values from instantaneous frequency components in the ST spread to a band area centered on the instantaneous frequency, leading to energy leakage [9]. This phenomenon leads to spectral energy distributions in locations where no energy should be present. Consequently, such false spectral energy diminishes the resolution of the ST and distorts the T-F distribution, impairing the precision and accuracy of GPR data processing.

To address energy leakage in T-F spectra, Daubechies et al. [10] introduced the synchrosqueezing transform (SST), which is based on the CWT. The SST enhances spectral resolution by concentrating dispersed energy in T-F spectra onto the instantaneous frequency. Over the past decade, researchers have successfully applied the SST in diverse fields, including paleoclimate change research [11], electrocardiography signal analysis [12,13], mechanical fault diagnosis [14,15,16], and signal denoising [17,18,19]. In geophysics, Wang et al. [20] utilized the SST on seismic data to reveal clearer channel features and more nuanced fault structures. Herrera et al. [21] found frequency slices of the SST and signal reconstruction error superior to traditional methods and used the SST to identify body waves in microseismic data with overlapping time phases [22]. Additionally, Mousavi et al. [23] employed the SST for denoising seismic signals and improving event detection and onset time estimation.

Huang et al. [24] introduced the synchrosqueezing S-transform (SS-ST), which achieved higher resolution than the SST and excelled at identifying gas hydrates through marine seismic data decomposition. Building on this, Wang et al. [25] and Tao et al. [26] proposed the synchrosqueezing generalized S-transform (SS-GST) based on the generalized S-transform (GST) [27,28,29], further enhancing T-F resolution and showing effectiveness at seismic data processing. However, the ST and the synchrosqueezing strategy are not fully compatible. The effectiveness of synchrosqueezing hinges on the T-F phase spectrum remaining constant over a frequency: a condition not met by the frequency-dependent phase spectrum of the ST and GST. Consequently, the SS-ST and SS-GST rely on the absolute values of the T-F spectrum as an alternative. This workaround results in diminished T-F resolution alongside reduced accuracy and speed in signal reconstruction. For dense GPR detection data, which are particularly vulnerable to electromagnetic environmental interference, maintaining a high-resolution T-F spectrum and swift signal reconstruction are imperative.

Addressing the spectral energy leakage in the ST and its incompatibility with the synchrosqueezing strategy, this paper introduces the synchrosqueezing generalized phase-shifting S-transform (SS-GPST). Based on the ST, the SS-GPST can be viewed as a three-step method. Initially, the ST undergoes phase-shifting to produce the phase-shifting S-transform (PST). The PST retains the ST amplitude spectrum and features the frequency-invariant phase spectrum, which ensures accuracy when overlaying complex spectra through the synchrosqueezing strategy. Subsequently, a generalization parameter enhances the flexibility of the PST spectrum. Finally, synchrosqueezing concentrates T-F energy on the instantaneous frequency. The phase-shifting addresses compatibility issues between the ST and synchrosqueezing, while the combined use of a generalization parameter and synchrosqueezing enhances T-F resolution. The SS-GPST retains the practicality of the ST for GPR processing and, notably, offers a highly focused and high-resolution T-F spectrum. Additionally, its inverse transform facilitates precise and rapid signal reconstruction.

In GPR tunnel detection data processing, we assessed the adaptability of the SS-GPST from three aspects: spectral energy distribution, thin layer identification, and denoising. The results indicate: (1) compared to other methods, the SS-GPST accurately expresses spectral components with a strong focusing degree and fewer interference components; (2) high-frequency slices of the SS-GPST accurately detect the top and bottom interfaces of a 3.0–3.5 cm reinforcement protection layer; and (3) due to fewer interference components in the SS-GPST spectrum, reconstructing GPR profiles through the SS-GPST inverse transform is an efficient denoising technique.

## 2. Basic Principles of the SS-PGST

**Figure 1:**
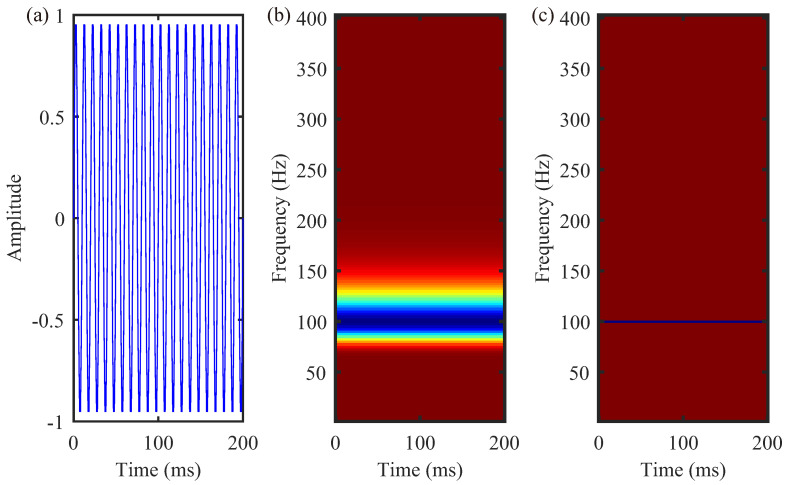
(**a**) Signal h(t)=cos(2π100t), (**b**) the ST of h(t), and (**c**) the SS-GPST of h(t).

The S-transform (ST) of a time-series signal h(t) is

(1) $$ST\left(\tau ,f\right)=\int_{-\infty }^{\infty }h\left(t\right)\frac{\left|f\right|}{\sqrt{2\pi }}e^{-\frac{{\left(t-\tau \right)}^{2}f^{2}}{2}}e^{-i2\pi ft}dt,$$

where *t* and *f* represent time and frequency variables, respectively, and τ is an additional time variable that defines the position of the window function over time. To motivate the idea of “synchrosqueezing”, consider a purely harmonic h(t)=cos(2π100t), as depicted in Figure 1a, where the spectral energy should theoretically concentrate at $f_{0}=100$ Hz. However, in the ST, spectral energy leaks around the instantaneous frequency $\tilde{f}=f_{0}$, causing false T-F distributions, as illustrated in Figure 1b. The aim of synchrosqueezing is to reconcentrate the T-F spectrum to the true instantaneous frequency of 100 Hz, achieving the more accurate representation shown in Figure 1c. In the ST spectrum, for any coordinates (τ,f), where ST(τ,f)≠0, the corresponding instantaneous frequency $\tilde{f}$ is:

(2) $$\tilde{f}\left(\tau ,f\right)=f+\frac{1}{2\pi iST(\tau ,f)}\frac{\partial ST(\tau ,f)}{\partial \tau }.$$

**Figure 2:**
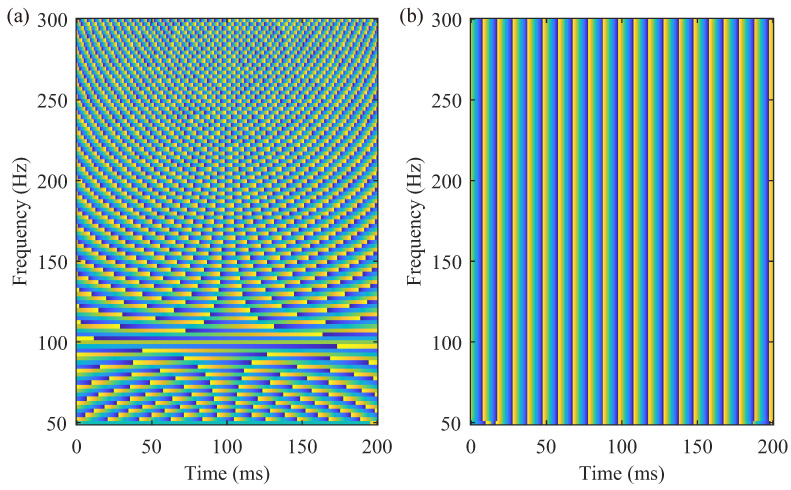
Phase spectrum of h(t)=cos(2π100t) in (**a**) ST and (**b**) PST. Different colors represent different phases, and (**b**) shows the phase that is invariant along the frequency (vertical) axis.

After obtaining the instantaneous frequency $\tilde{f}\left(\tau ,f\right)$ at each location (τ,f) on the ST spectrum, the subsequent step, known as synchrosqueezing, compresses the ST coefficient towards the closest instantaneous frequency. Equation (2), which defines the instantaneous frequency in terms of *f*, illustrates that the phase spectrum of the ST (Figure 2a) varies with *f*. The changing spectrum phase can lead to the cancellation of complex numbers ST(τ,f) when summed, highlighting the partial incompatibility between the ST and the synchrosqueezing strategies. To overcome this limitation, we propose the phase-shifting S-transform (PST), which entails a shift in the phase of *f*. The PST is delineated as follows:

(3) $$\begin{array}{cc} PST(\tau ,f) & =e^{i2\pi f\tau }ST\left(\tau ,f\right)\\ \; & =\int_{-\infty }^{\infty }h\left(t\right)\frac{\left|f\right|}{\sqrt{2\pi }}e^{\frac{-{\left(t-\tau \right)}^{2}{\left(f\right)}^{2}}{2}}e^{-i2\pi f(t-\tau )}dt. \end{array}$$

The PST retains the same T-F amplitude as the ST because multiplying the ST by $e^{i2\pi f\tau }$ does not change the amplitude spectrum. However, as demonstrated in Figure 2b, the critical distinction lies in the phase spectrum of the PST being independent of the frequency *f*. Phase-shifting harmonizes the compatibility between synchrosqueezing and the ST.

The generalized S-transform (GST) is an optimization of the ST and can improve the T-F resolution. To optimize the T-F resolution of the PST, we introduce a generalization parameter to the PST to control the shape of the Gaussian window. The generalized phase-shifting S-transform (GPST) is expressed as

(4) $$GPST\left(\tau ,f\right)=\int_{-\infty }^{\infty }h\left(t\right)\frac{\left|Af\right|}{\sqrt{2\pi }}e^{\frac{-{\left(t-\tau \right)}^{2}{\left(Af\right)}^{2}}{2}}e^{-i2\pi f(t-\tau )}dt,$$

where *A* is a positive generalization parameter. In the GPST, the instantaneous frequency $\bar{f}$ of GPST is

(5) $$\bar{f}\left(\tau ,f\right)=\frac{1}{2\pi iGPST(\tau ,f)}\frac{\partial GPST(\tau ,f)}{\partial \tau }.$$

Equation (5) indicates that $\bar{f}$ is constant in the frequency direction and that the GPST is perfectly suited for synchrosqueezing. The derivation of Equation (5) is presented in Appendix A. Based on the instantaneous frequency $\bar{f}$, the synchrosqueezing generalized phase-shifting S-transform (SS-GPST) can be defined as

(6) $$SS-GPST\left(\tau ,{\bar{f}}_{l}\right)=\sum_{f_{k}:\left|\bar{f}\left(\tau ,f_{k}\right)-{\bar{f}}_{l}\right|\leq \Delta \bar{f}/2}GPST\left(\tau ,f_{k}\right){f_{k}}^{-1},$$

where $f_{k}$ is the discrete frequency, and ${\bar{f}}_{l}$ and $\Delta \bar{f}$ are the central frequency and the frequency interval, respectively, of the SS-GPST spectrum. An SS-GPST spectrum with 100 Hz harmonics is shown in Figure 1c. The analyzed signal can be reconstructed from the SS-GPST by

(7) h(t)=ReC−1∑lSS−GPST(τ,f¯l)Δf¯,

where $C=-A/2\int_{0}^{\infty }\hat{\omega }\left(\xi \right)\xi^{-1}d\xi$, $\omega \left(t\right)=\left(1/\sqrt{2\pi }\right)e^{\left(-t^{2}A^{2}/2\right)}e^{i2\pi t}$, and $\hat{\omega }\left(\xi \right)$ is the Fourier transform of ω(t). Details of the derivation of Equation (7) can be found in Appendix B. According to the properties of the Gaussian function, we also derive the approximate reconstruction formula of the SS-GPST in Appendix C.

(8) h(τ)≈Re22πA∑lSS−GPST(τ,f¯l)Δf¯.

In Equation (8), the approximate inverse transform of the SS-GPST is primarily based on summation, indicating a straightforward and rapid signal reconstruction process. Within the SS-GPST, phase-shifting serves as the initial step to improve compatibility between the ST and synchrosqueezing, and then, the incorporation of a generalization parameter enhances the transform’s adaptability and resolution. Finally, synchrosqueezing focuses the T-F spectrum precisely on the instantaneous frequency, thereby optimizing the accuracy of spectral analysis.

## 3. Synthetic Data Test

To illustrate the performance of the SS-GPST in the TF spectrum and signal reconstruction, we apply the continuous wavelet transform (CWT), S-transform (ST), generalized S-transform (GST), synchrosqueezing transform (SST), synchrosqueezing S-transform (SS-ST), synchrosqueezing generalized S-transform (SS-GST), and SS-GPST on a synthetic signal. The analyzed signal s(t) is the sum of three different components, the mathematical expressions of which are as follows:

(9) $$\begin{array}{cc} s(t) & =s_{1}\left(t\right)+s_{2}\left(t\right)+s_{3}\left(t\right),\\ s_{1}\left(t\right) & =[1.8+\cos(t)]\cdot \cos[2\pi (3t+0.5\cos(t))],\\ s_{2}\left(t\right) & =0.7\left[1+0.3\cos\left(2t\right)\right]\cdot e^{-t/20}\cdot \cos\left[2\pi \left(3t+0.6t^{2}+0.8\sin\left(t\right)\right)\right],\\ s_{3}\left(t\right) & =0.5\cos[2\pi (9t)]. \end{array}$$

**Figure 3:**
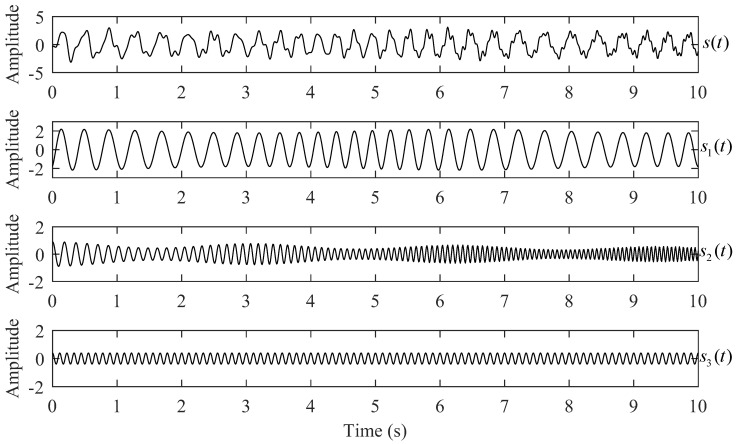
Synthetic signal s(t) and its three different components.

Figure 3 shows the synthetic signal and its three components, each with 1024 sampling points, over a time range t∈[0,10]. Each of the three components exhibits unique characteristics: $s_{1}\left(t\right)$ features the lowest frequency and largest amplitude, whereas the amplitude of $s_{2}\left(t\right)$ diminishes with increasing frequency; $s_{3}\left(t\right)$ is a 9 Hz cosine signal characterized by the smallest amplitude. The synthetic signal has typical geophysical characteristics, wherein high-frequency components typically have low amplitudes in T-F spectra.

### 3.1. Comparison of Time–Frequency Spectra Using Different Methods

**Figure 4:**
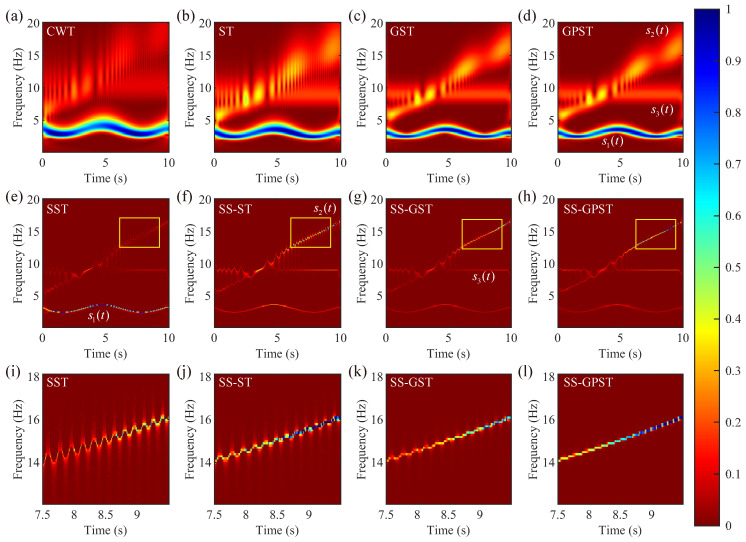
Normalized T-F spectra of the synthetic signal by different methods: (**a**) CWT, (**b**) ST, (**c**) GST, (**d**) GPST, (**e**) SST, (**f**) SS-ST, (**g**) SS-GST, and (**h**) SS-GST. We zoom the results (yellow boxs) of (**i**) SST, (**j**) SS-ST, (**k**) SS-ST, and (**l**) SS-GPST. Generalization parameter A=0.68 in the GST, GPST, SS-GPST, and SS-GPST.

Drawing on previous studies [11,24] and after comparing various wavelet types, we selected the Morlet wavelet as the mother wavelet for the CWT and SST. Figure 4 presents normalized T-F spectra of the synthetic data. Figure 4a–d illustrate T-F spectra of the synthetic signal obtained using the CWT, ST, GST, and PGST. Relative to the CWT, the ST better highlights high-frequency signals, and both the GST and GPST further improve the T-F resolution of the ST. As phase-shifting does not change the T-F spectrum energy of the GST, Figure 4c,d show that the GPST and GST have the same distribution. However, the above T-F transforms inevitably leak spectral energy into the band-shaped regions centered around the instantaneous frequency, causing false T-F distributions and demonstrating lower resolution, as shown in Figure 4a–d.

Conversely, synchrosqueezing significantly improves the T-F resolution of three components in the SST, SS-ST, SS-GST, and SS-GPST, as illustrated in Figure 4e–h. Within these synchrosqueezing methods, spectral energy is focused on the three principal components of T-F spectra. However, synchrosqueezing methods vary in their level of focus and representation of components. The SST excels at isolating the low-frequency component $s_{1}\left(t\right)$ but is less effective for $s_{2}\left(t\right)$ and $s_{3}\left(t\right)$. Methods based on the ST are predominantly better at analyzing high-frequency components. However, the partial incompatibility between the ST and synchrosqueezing leads to energy dispersion and reduced resolution in both the SS-ST and SS-GST. For instance, with the fixed 9 Hz signal $s_{3}\left(t\right)$, the SS-ST and SS-GST produce imprecise outcomes, in contrast to the superior accuracy of the SS-GPST. The analysis of higher-frequency components $s_{2}\left(t\right)$ via local magnification is shown in Figure 4i–l. Here, the SST is observed to underperform with high-frequency components, while the SS-ST and SS-GST enhance T-F resolution. The compatibility between the ST and synchrosqueezing, achieved through phase-shifting, endows the SS-GPST with the highest focusing efficacy and resolution, showcasing its advantage in detailed signal analysis.

### 3.2. Reconstruction Analysis

To evaluate the signal reconstruction error of different T-F transforms, we employ two metrics: maximum error $E_{\max}$ and mean squared error (MSE). They are defined as follows:

(10) $$\begin{array}{cc} E[n] & =s\left[n\right]-s^{\prime }\left[n\right],n=1,2,3,\cdot \cdot \cdot ,N,\\ E_{\max} & =\max(\left|E[n]\right|),\\ \mathrm{MSE} & =\frac{1}{N}\sum_{i=1}^{N}{\left(s\left[n\right]-s^{\prime }\left[n\right]\right)}^{2}, \end{array}$$

where s[n] is the original signal, and $s^{\prime }\left[n\right]$ is the reconstructed signal of T-F spectra.

**Figure 5:**
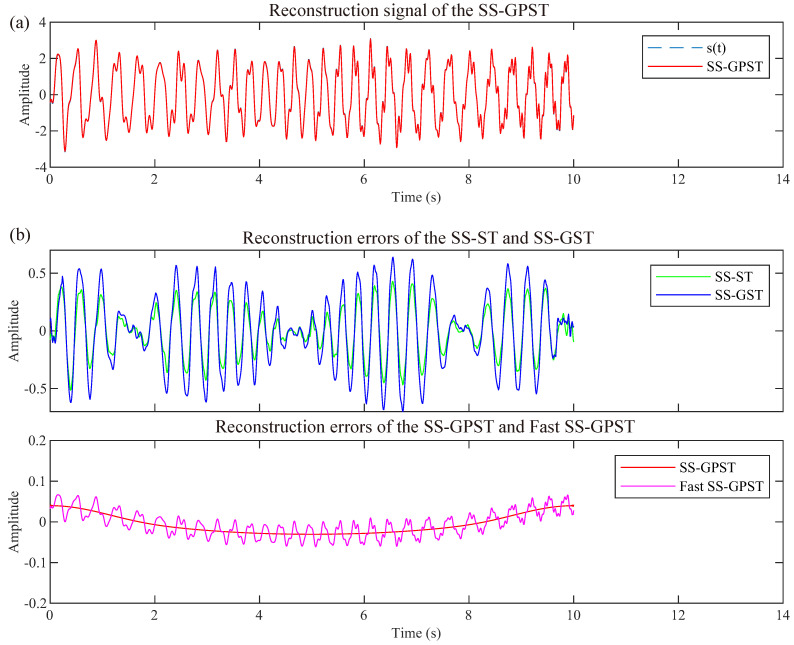
(**a**) Reconstructed signal of the SS-GPST; (**b**) error *E* of the SS-ST, SS-GST, and SS-GPST.

As demonstrated in Figure 5a, the SS-GPST signal reconstruction aligns closely with the original synthetic signal, indicating that the SS-GPST inverse transform accurately reconstructs the analyzed signal. Furthermore, Figure 5b illustrates a comparison of reconstruction errors between the SS-ST, SS-GST, SS-GPST, and Fast SS-GPST. Figure 5b reveals that the incompatibility between the ST and synchrosqueezing leads to notable reconstruction errors in the SS-ST and SS-GST, particularly for the SS-GST, where the maximum error approaches 0.70. In contrast, the SS-GPST and Fast SS-GPST show minimal reconstruction errors (less than 0.1), with the SS-GPST utilizing Equation (7) for calculation and the Fast SS-GPST employing Equation (8).

Table 1 presents quantitative metrics for the synthetic signal reconstruction. The SS-ST and SS-GST show significant errors, whereas the SS-GPST and Fast SS-GPST demonstrate smaller errors. Despite the SST having the smallest error, the superior T-F resolution of the SS-GPST renders it more suitable for processing high-frequency, low-amplitude signals in GPR data. In terms of reconstruction time, the Fast SS-GPST is notably quicker than the other methods; this is attributed to its simpler inverse transform. This efficiency underscores the practical value of the SS-GPST in applications.

This section compares the performance of different T-F transforms on the synthetic data and concludes that: (1) The SS-GPST achieves superior T-F spectrum focusing and resolution, ensuring accurate component representation. (2) The SS-GPST inverse transform reconstructs the analyzed signal with low error and high efficiency.

## 4. GPR Real Data Test

T-F transforms are essential in GPR data processing. This section uses a GPR tunnel detection task as a case study to evaluate the efficacy of the SS-GPST across three dimensions: T-F spectral energy distribution, thin layer identification, and data denoising. Section 4.1 details the characteristics of GPR data and methods for extracting high-resolution T-F spectra. Section 4.2 discusses the effectiveness of the SS-GPST at analyzing the T-F spectral energy distribution. Section 4.3 delves into the analysis of frequency slices for extracting the thin reinforcement protection layer. Section 4.4 demonstrates the denoising capability of the SS-GPST through signal reconstruction.

### 4.1. GPR Data and High-Resolution Time–Frequency Spectra Extraction

**Figure 6:**
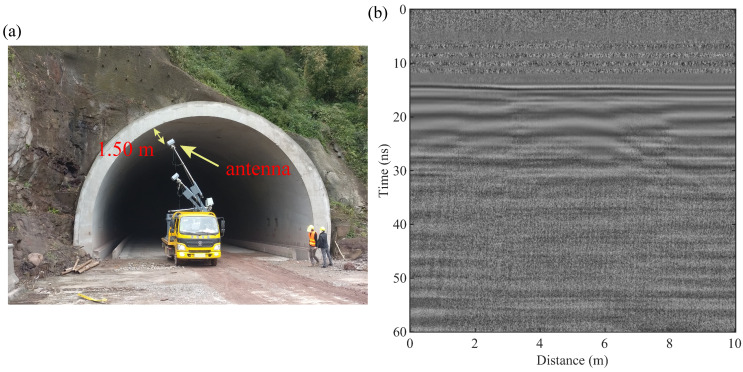
(**a**) Inspection site photo; (**b**) raw GPR data.

As depicted in Figure 6, the raw GPR data were recorded by a vehicle-mounted GPR system [30] equipped with 300 MHz air-coupled antennas to measure a highway tunnel in China. The antennas cover a frequency band ranging from 150 MHz to 500 MHz. Figure 6a presents a photograph of the on-site inspection and shows the antennas positioned approximately 1.50 m from the tunnel lining. During the data acquisition phase, the time window was set to 60 ns, and trace spacing was maintained at 0.01 m. The primary objectives of our detection effort were to ascertain the thickness of the reinforcement protection layer and the lining layer in addition to evaluating the internal condition of the lining. Figure 6b illustrates a segment of the original data after applying a process to remove the global mean background, which enhances the clarity of the data for analysis.

**Figure 7:**
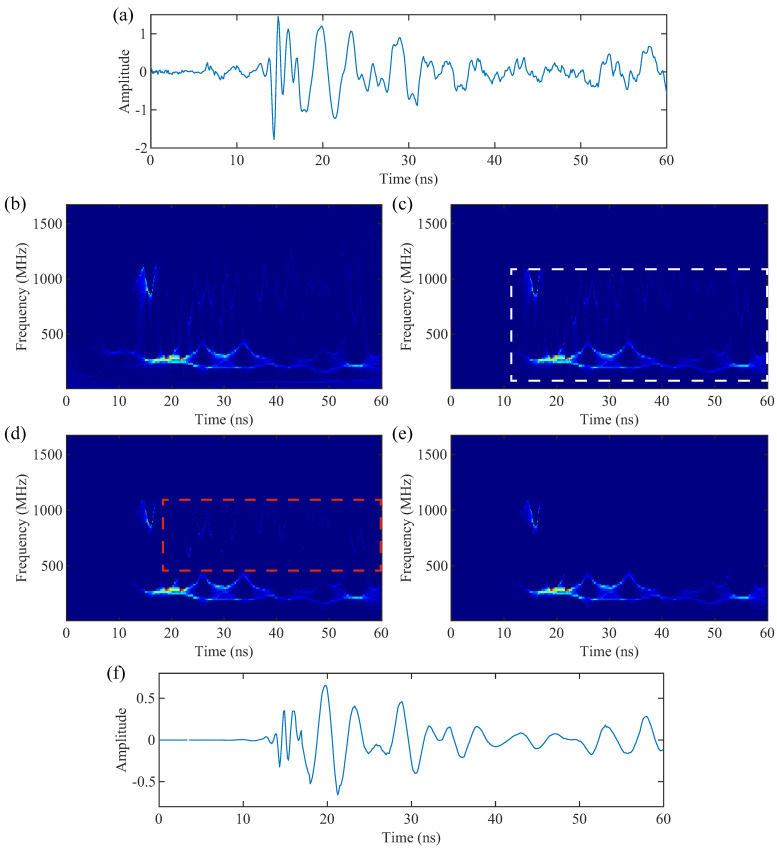
High-resolution T-F spectra acquisition process. (**a**) Average trace of the raw data, (**b**) T-F spectrum of the SS-GPST, (**c**) the region of interest in the T-F spectrum, (**d**) hard threshold processing, (**e**) pixel connectivity threshold processing, and (**f**) reconstructed signal. The white dashed box represents the approximate range of spectral energy, and the red dashed box indicates the range of major interference components in the T-F spectrum.

Traditional T-F analysis methods, such as the CWT and ST, often struggle with spectral leakage in their T-F spectra, wherein effective signals and interference signals intermingle. This issue makes it challenging to achieve high-resolution and focused T-F spectra. However, the synchrosqueezing concentrates the diffused spectral energy onto the instantaneous frequency, significantly improving T-F resolution and separating effective signals from interference. We propose a four-step high-resolution T-F spectra extraction method for GPR data. Taking the average of all A-scans from the raw data as an example, Figure 7 demonstrates the process of acquiring high-resolution TF spectra:

(1) The SS-GPST transforms A-scan data into the T-F domain, producing a preliminarily focused T-F spectrum, as shown in Figure 7b. It is then necessary to separate the interference signals from the SS-GPST result and retain the useful signals.
(2) We roughly select the region of the effective signal in the T-F spectrum. As depicted in Figure 7c, the selected region (within the white dashed box) encompasses the lining and rock layers and has a time range of 12–60 ns and a frequency range of roughly 100–1100 MHz.
(3) Hard threshold filtering converts the interference components of the T-F spectrum into discrete noise points, as illustrated by the red dashed box in Figure 7d.
(4) Pixel connectivity threshold filtering is then utilized to remove isolated noise points since most of the interference energy has become isolated noise points (shown in Figure 7d). Figure 7e demonstrates that this method effectively preserved information in large connected regions of the T-F spectra while eliminating poorly connected interference. Details on pixel connectivity filtering can be found in [31].

Finally, Figure 7f presents the results from the SS-GPST inverse transform. A comparison between Figure 7a,f clearly shows that the signals reconstructed with high-resolution T-F spectra have enhanced smoothness and reduced interference signals.

### 4.2. Time–Frequency Spectral Energy Distribution

**Figure 8:**
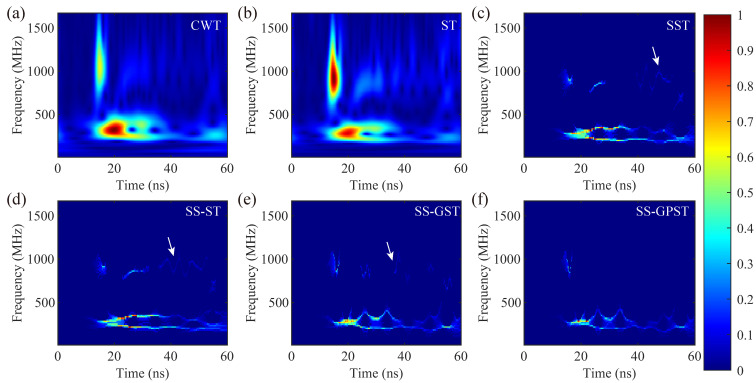
Normalized T-F spectra of different methods: (**a**) CWT, (**b**) ST, (**c**) SST, (**d**) SS-ST, (**e**) SS-GST, and (**f**) SS-GPST.

The T-F spectra of the average A-scan (Figure 7a) are presented in Figure 8, where the SST, SS-ST, SS-GST, and SS-GPST all employ the high-resolution T-F spectral extraction method demonstrated in Section 4.1. The test GPR tunnel detection data were recorded using a 300 MHz antenna. Effective components in T-F spectra should include the main frequency energy between 100–500 MHz and the high-frequency energy of the reinforcement protection layer on the lining surface.

Figure 8a,b illustrate traditional CWT and ST results and display both the main frequency band and reinforcement protection layer energy (around 1000 MHz) but with low T-F spectral resolution and noticeable interference (500–1500 MHz) within 20–60 ns. These interference components may originate from internal device noise, environmental electromagnetic signals, multiple reflections near the antenna, and/or improper data preprocessing. For the main frequency band components between 15–60 ns, the frequency distribution of the SST and SS-ST spans 180–350 MHz for main band components (15–60 ns), which is lower than expected. In contrast, the SS-GST and SS-GPST, covering 150–450 MHz, align more closely with the anticipated 100–500 MHz range, showcasing superior performance. In T-F components above 500 MHz, while all synchrosqueezing methods retained the reinforcement protection layer components near 800–1100 MHz at 15 ns, limited resolution and focusing led to residual interference components (white arrows) distributed from 20–60 ns, as shown in Figure 8c–e. In contrast, the SS-GPST effectively isolated interference energy in high-resolution T-F extraction, as demonstrated in Figure 8f. The SS-GPST delivers a highly focused and high-resolution T-F spectrum that accurately represents T-F components without interference, markedly surpassing other T-F techniques.

### 4.3. Time–Frequency Slice Analysis for the Reinforcement Protection Layer

Measuring the thickness of the reinforcement protection layer is a crucial task in tunnel inspections. The test section of the tunnel features a reinforced concrete lining with a 20 × 20 cm mesh grid size that is constructed from HRB400 grade steel rebar with a diameter of 22 mm. The reinforcement protection layer, designed to be 3.0–4.0 cm, acts as a crucial barrier between the rebar mesh and the lining surface, safeguarding the rebar from environmental exposure and enhancing the structural integrity of the tunnel. However, identifying layers thinner than 1/4 of the wavelength (approximately 8.8 cm) in the time-domain profiles of a 300 MHz antenna poses significant challenges. Nonetheless, ultra-wideband GPR antennas, which emit high-frequency energy beyond 300 MHz, facilitate the identification of such thin layers in shallow locations with minimal signal attenuation via T-F analysis. For instance, the ST has been used successfully to identify thin sediment layers in GPR studies [7]. Given that the reinforcement protection layer is located on the surface of the lining, closest to the antenna, it is feasible to identify this thin layer through T-F slices.

**Figure 9:**
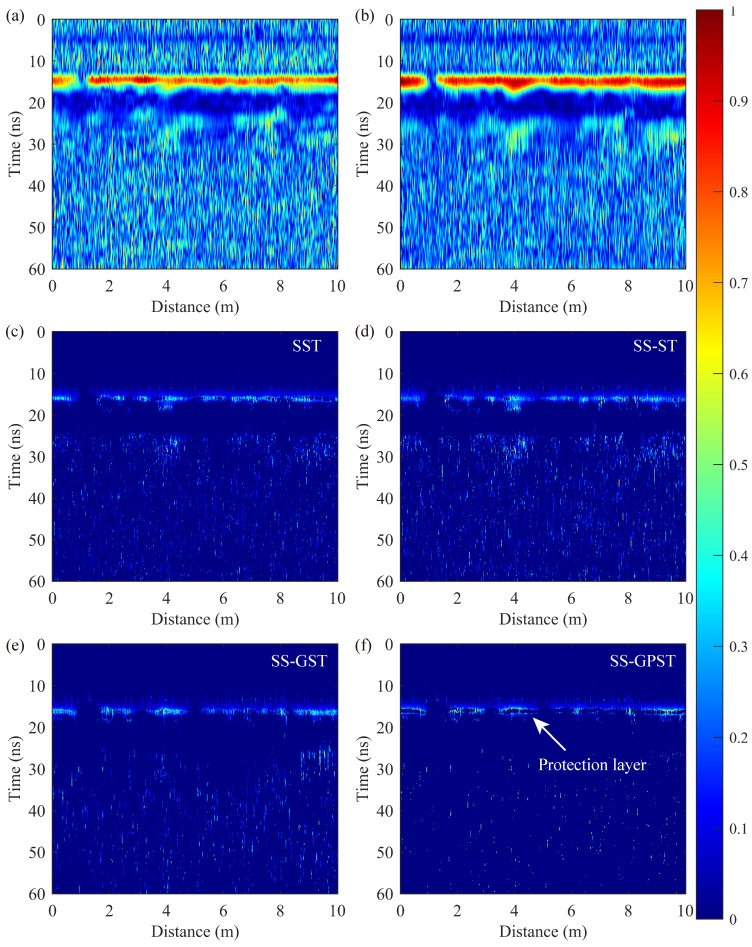
Normalized 900 MHz slices of different methods: (**a**) CWT, (**b**) ST, (**c**) SST, (**d**) SS-ST, (**e**) SS-GST, and (**f**) SS-GPST.

Figure 8 reveals the reinforcement protection layer within T-F spectra at 800–1100 MHz, so we select 900 MHz slices as the analysis object. As shown in Figure 9, the reinforcement protection layer near 15 ns is reflected in T-F slices, but the ST, CWT, SST, SS-ST, and SS-GST are unable to accurately identify the thin layer due to limited focus and resolution. Conversely, the SS-GPST accurately delineates the upper and lower boundaries of the reinforcement protection layer, confirming a thickness of 3.0–3.5 cm, which aligns with design specifications.

Moreover, frequency slice analysis indicates that the ST and CWT allow high-frequency noise to pervade the entire GPR profile. On the other hand, synchrosqueezing methods concentrate high-frequency noise into discrete points, with the SS-GPST showing the least number of noise points. This distinction further highlights the superior performance of SS-GPST in T-F analysis.

### 4.4. GPR Profile Reconstruction

A crucial application of T-F transforms in GPR data processing is denoising by filtering out interference from T-F spectra, especially for tasks that require denoising or preserving specific components. For GPR tunnel detection tasks, accurately determining the thickness of linings and reinforcement protection layers is essential. This work requires both denoising the data and preserving high-frequency surface reflections. As outlined in Section 4.1, the process of extracting synchrosqueezing T-F spectra has effectively suppressed interfering components, rendering the reconstruction of the GPR profile equivalent to data denoising.

**Figure 10:**
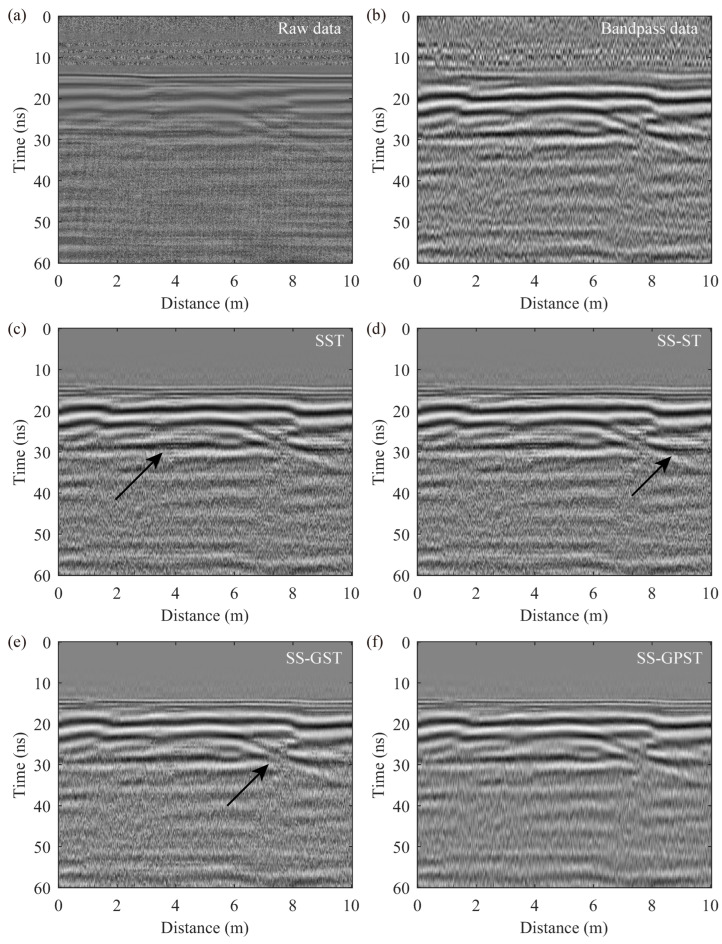
GPR profile reconstruction results using different methods: (**a**) raw data, (**b**) band-pass filtering, (**c**) SST, (**d**) SS-ST, (**e**) SS-GST, and (**f**) SS-GPST. Black arrows indicate the subtle high-frequency interference components.

Figure 10 compares various synchrosqueezing transforms with band-pass filtering (preserving 100–500 MHz). The band-pass filter is applied across the time window and suppresses all high-frequency GPR signals, obscuring crucial surface details and complicating measurements of lining thickness and reinforcement protection layer thickness, as shown in Figure 10b. In contrast, Figure 10c–f demonstrate that synchrosqueezing methods effectively retain high-frequency signals around 15 ns. This retention makes surface reflections distinctly observable, pinpointing the lining layer within the 15–29 ns range. The T-F analyses of Section 4.2 and Section 4.3 show that the SS-GPST has minimal interference, resulting in a nearly noise-free high-frequency reconstruction profile in Figure 10f, in contrast to the marked interference (black arrows) observed in Figure 10c–e. The results from Figure 10 suggest that the SS-GPST excels at both preserving critical high-frequency surface signals and suppressing internal high-frequency interference, surpassing alternative methods.

**Figure 11:**
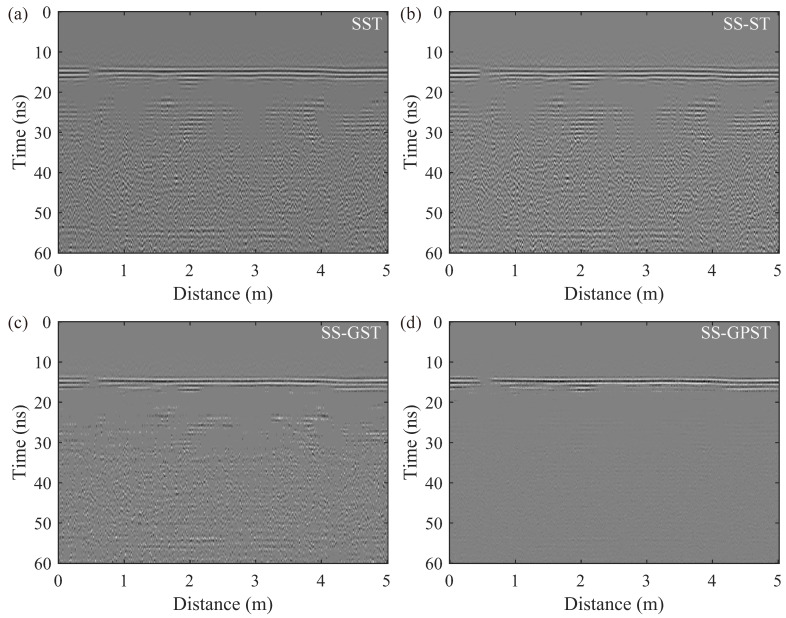
Residual noise of the (**a**) SST, (**b**) SS-ST, (**c**) SS-GST, and (**d**) SS-GPST at 500–1000 MHz.

To better highlight the differences in suppressing interference, Figure 11 extracts the high-frequency components (500–1000 MHz) from the reconstructed profiles of various synchrosqueezing transforms displayed in Figure 10c–f. Figure 11 shows that the strong energy reflections between 10–20 ns stem from the tunnel surface and the reinforcement protection layer. In the remaining sections of the profile, the SS-GPST displays minimal interference components, demonstrating its effectiveness at suppressing most interference within its high-resolution T-F spectrum.

## 5. Conclusions

To address the issue of spectral energy leakage in the S-transform (ST) during ground penetrating radar (GPR) data processing, this study introduces the synchrosqueezing generalized phase-shifting S-transform (SS-GPST) along with its forward, inverse, and approximate inverse transformations. The SS-GPST employs phase-shifting to resolve compatibility issues between the ST and synchrosqueezing and utilizes a generalization parameter and synchrosqueezing to achieve enhanced focus and resolution in the time–frequency (T-F) spectrum. Testing with a tri-component synthetic signal demonstrates that the SS-GPST outperforms other synchrosqueezing methods in terms of focusing degree, resolution, reconstruction error, and speed. For actual GPR data, we propose a scheme to obtain high-resolution T-F spectra and evaluate the adaptability of the SS-GPST in a tunnel detection task that includes spectral energy distribution, thin layer identification, and denoising. The results of GPR data processing indicate:

(1) Compared to other methods, the SS-GPST accurately expresses spectral components with a strong focusing degree and fewer interference components.
(2) High-frequency slices from the SS-GPST can accurately depict the upper and lower interfaces of the reinforcement protection layer and accurately measure thickness at 3.0–3.5 cm.
(3) Due to fewer interference components in the SS-GPST spectrum, reconstructing GPR profiles through the SS-GPST inverse transform is an efficient denoising technique.

The SS-GPST has been integrated into our open-source platform, GPRlab [32], to serve as a new tool for GPR data processing and replacing the traditional ST approach. We plan to validate the generalizability of the SS-GPST in more case studies. A limitation of this study is the restricted practical testing of the SS-GPST. In future work, we aim to validate the generalizability of the SS-GPST across additional case studies. 

## Figures and Tables

**Table 1:** Signal reconstruction indexes for different T-F methods.

Index	SST	SS-ST	SS-GST	SS-GPST	Fast SS-GPST
$E_{\max}$	0.03	0.51	0.70	0.04	0.07
MSE	1.97 × 10^−4^	0.04	0.10	6.00 × 10^−4^	9.46 × 10^−4^
Time (s)	0.25	0.28	0.54	0.22	2.49 × 10^−3^

## Data Availability

The data and code are available at https://github.com/xiongGPR (accessed on 1 April 2023).

## Appendix A. Derivation of Instantaneous Frequency in GPST

The GPST of a signal h(t) is defined by Equation (7):

(A1) $$GPST\left(\tau ,f\right)=\int_{-\infty }^{\infty }h\left(t\right)\frac{\left|Af\right|}{\sqrt{2\pi }}e^{\frac{-{\left(t-\tau \right)}^{2}{\left(Af\right)}^{2}}{2}}e^{-i2\pi f(t-\tau )}dt,$$

where *A* is a constant. Let $\omega \left(t\right)=\left(1/\sqrt{2\pi }\right)e^{\left(-t^{2}A^{2}/2\right)}e^{-i2\pi t}$. Equation (A1) can then be expressed as

(A2) $$GPST\left(\tau ,f\right)=\int_{-\infty }^{\infty }h(t)\left|Af\right|\omega \left[f\left(t-\tau \right)\right]dt.$$

According to Plancherel’s theorem, Equation (A2) can be written as

(A3) $$GPST\left(\tau ,f\right)=\frac{\left|A\right|}{2\pi }\int_{-\infty }^{\infty }\hat{h}\left(\xi \right)\hat{\omega }\left(f^{-1}\xi \right)e^{i\tau \xi }d\xi ,$$

where ξ is the frequency, $\hat{h}\left(\xi \right)$ is the Fourier transform of h(t), and $\hat{\omega }\left(\xi \right)$ is the Fourier transform of ω(t). If the signal has the form of $h\left(t\right)=M\cos\left(2\pi f_{0}t\right)$, for which the Fourier transform is $\hat{h}\left(\xi \right)=M\pi \left[\delta \left(\xi -2\pi f_{0}\right)+\delta \left(\xi +2\pi f_{0}\right)\right]$, Equation (A3) becomes

(A4) $$GPST\left(\tau ,f\right)=\frac{\left|A\right|M}{2}\hat{\omega }\left(f^{-1}2\pi f_{0}\right)e^{i\tau 2\pi f_{0}}.$$

Since $\left(\partial /\partial \tau \right)GPST\left(\tau ,f\right)=i\pi f_{0}\left|A\right|M\hat{\omega }\left(f^{-1}2\pi f_{0}\right)e^{i\tau 2\pi f_{0}}$, the instantaneous frequency of the signal, $\bar{f}$, can be calculated by

(A5) $$\bar{f}\left(\tau ,f\right)=\frac{1}{2\pi iGPST(\tau ,f)}\frac{\partial GPST(\tau ,f)}{\partial \tau }.$$

## Appendix B. Derivation of Inverse SS-GPS

The definition of the SS-GPST is given by

(A6) $$SS-GPST\left(\tau ,{\bar{f}}_{l}\right)=\sum_{f_{k}:\left|\bar{f}\left(\tau ,f_{k}\right)-{\bar{f}}_{l}\right|\leq \frac{\Delta \bar{f}}{2}}GPST\left(\tau ,f_{k}\right){f_{k}}^{-1}.$$

The following argument shows that the signal can still be reconstructed after the synchrosqueezing. Considering Equation (A3), we have

(A7) $$\begin{array}{cc} \int_{0}^{\infty }GPST\left(\tau ,f\right)f^{-1}df & =\int_{0}^{\infty }\frac{\left|A\right|}{2\pi }\int_{-\infty }^{\infty }\hat{h}\left(\xi \right)\hat{\omega }\left(f^{-1}\xi \right)e^{i\tau \xi }f^{-1}d\xi df\\ \; & =-\frac{\left|A\right|}{2\pi }\int_{0}^{\infty }\int_{0}^{\infty }\hat{h}\left(\xi \right)\hat{\omega }\left(\zeta \right)e^{i\tau \xi }\frac{1}{\zeta }d\xi d\zeta \\ \; & =\frac{1}{\pi }\int_{0}^{\infty }\hat{h}\left(\xi \right)e^{i\tau \xi }d\xi \cdot \left(-\frac{\left|A\right|}{2}\right)\int_{0}^{\infty }\hat{\omega }\left(\zeta \right)\frac{1}{\zeta }d\zeta . \end{array}$$

Setting $C=-A/2\int_{0}^{\infty }\hat{\omega }\left(\xi \right)\xi^{-1}d\xi$ and considering the signal to be real, the signal can then be reconstructed by

(A8) h(τ)=π−1Re∫0∞h^(ξ)eiτξdξ=ReC−1∫0∞GPST(τ,f)f−1df.

In the piecewise constant approximation corresponding to the binning in *f*, Equation (A8) becomes

(A9) h(τ)≈ReC−1∑kGPST(τ,fk)fk−1(Δf)k=ReC−1∑lSS−GPST(τ,fl)Δf¯.

## Appendix C. Derivation of Approximate Inverse SS-GPST

The GPST of a signal h(t) is defined by Equation (7):

(A10) $$GPST\left(\tau ,f\right)=\int_{-\infty }^{\infty }h\left(t\right)\frac{\left|Af\right|}{\sqrt{2\pi }}e^{\frac{-{\left(t-\tau \right)}^{2}{\left(Af\right)}^{2}}{2}}e^{-i2\pi f(t-\tau )}dt.$$

Setting $m\left(t,f\right)=\frac{\left|Af\right|}{\sqrt{2\pi }}e^{\frac{-{\left(t-\tau \right)}^{2}{\left(Af\right)}^{2}}{2}}$, then

(A11) $$\begin{array}{cc} \int_{-\infty }^{\infty }GPST(\tau ,f)df & =\int_{-\infty }^{\infty }\int_{-\infty }^{\infty }h\left(t\right)m\left(t-\tau ,f\right)e^{-i2\pi f(t-\tau )}dtdf\\ \; & \approx \int_{-\infty }^{\infty }h\left(t\right)m\left(t-\tau ,f\right)\int_{-\infty }^{\infty }e^{-i2\pi f(t-\tau )}dtdf\\ \; & \approx \int_{-\infty }^{\infty }h(t)m(t-\tau ,f)\delta (t-\tau )dt\\ \; & \approx h(\tau )m(0,f)\\ \; & \approx h\left(\tau \right)\frac{\left|Af\right|}{\sqrt{2\pi }}. \end{array}$$

Therefore, in the discrete calculation, the inverse transformation of the SS-GPST can be obtained as follows:

(A12) h(τ)≈Re22πA∑lSS−GPST(τ,f¯l)Δf¯.
